# Dietary supplements intake among youth: preliminary results of a multicentric survey

**DOI:** 10.1093/eurpub/ckac131.230

**Published:** 2022-10-25

**Authors:** C Protano, A De Giorgi, F Valeriani, F Gallè, G Liguori, VR Spica, M Vitali

**Affiliations:** 1 Public Health Department, Sapienza University of Rome, Rome, Italy; 2 Public Health Department, Foro Italico University, Rome, Italy; 3 Public Health Department, Parthenope University, Naples, Italy

## Abstract

**Background:**

In the last decades, a wide spread of dietary supplements (DSs) has been observed worldwide. However, DS use is not always motivated by real nutritional needs. In this context, commercial strategies and peers’ suggestions seem to play an important role in determining this habit, with harmful effects on consumers’ health. This cross-sectional study was aimed to evaluate the DS consumption patterns among a sample of undergraduate students attending 14 Italian universities.

**Methods:**

An anonymous web questionnaire was used to collect information about DS use and consumption habits, related motivations and possible adverse effects. The survey is still ongoing and its conclusion is expected to be on May 2022.

**Results:**

On a total of 2019 participants enrolled so far (69.7% female, mean age 22.8±4.7), 72.6% reported the use of at least a DS in the last six months. Multivitamin and multimineral products were the most used, being reported by 35.8% of the participants, while caffeinated energy supplements were the less reported (2.0%). The main reason for DS use was to deal with a specific deficiency following a physician or nutritionist indication (47.8%). The 3.4% of participants reported the occurrence of negative outcomes, mainly gastrointestinal disorders (74%).

**Conclusions:**

These preliminary results evidenced a wide use of DSs in the studied population. Although the main motivation was a nutritional need with a medical recommendation, a notable proportion of the sample assumed DSs without any specific prescription. Thus, it is essential to increase the knowledge about DSs and related threats deriving from their inappropriate use in the population, especially among youths. Further analysis will allow to identify possible correlations with socio-demographic and behavioural variables.

**Key messages:**

• It is essential to increase the knowledge about dietary supplements to avoid an inappropriate use in the population.

• Youths are the most exposed to this consumption.

